# A 2.5-cm single-port video-assisted thoracoscopic surgery for stage III tuberculous empyema: a case report

**DOI:** 10.1186/s13019-023-02116-5

**Published:** 2023-01-11

**Authors:** Zhi Yang, Xiujun Chang

**Affiliations:** Department of Thoracic Surgery, Beijing Chest Hospital Affiliated to Capital Medical University, Courtyard 9, Beiguan Street, Tongzhou District, Beijing, China

**Keywords:** Thoracoscopy, Video-assisted thoracoscopic surgery, Tuberculous empyema, Surgery/incisions

## Abstract

**Background:**

Stage III tuberculous empyema is a common disease of tuberculosis. Traditionally, it has been treated by thoracotomy or video-assisted thoracoscopic surgery with two to four incisions. But conventional surgery has large trauma, large bleeding volume and long recovery time. To our knowledge it is the first report of surgery for stage III tuberculous empyema with a mini single-port approach.

**Case presentation:**

A 23-year-old woman admitted to our hospital with complaints of intermittent chest pain for half a year. We got the diagnosis of stage III tuberculous empyema after medical treatment. Considering that the patient was young and unmarried, we decided to perform minimally invasive pleural decortication through a 2.5 cm single port. The operation time was 240 min, and blood loss was 100 ml. The patient recovered well and postoperative pain was mild.

**Conclusion:**

This case demonstrates that single-port VATS with a smaller incision for the Stage III tuberculous empyema should be considered in well selected patients.

Tuberculous empyema is a common disease of tuberculosis, which usually caused by improper treatment such as incomplete drainage of pleural effusion [1]. The development of tuberculous empyema progresses through three stages: an exudative stage (stage I), a fibrinopurulent stage (stage II), and an organizing or consolidation stage (stage III) [2]. When the disease develops into stage III empyema, and the drug treatment effect is poor, which requires surgical treatment. Traditionally, tuberculous empyema has been treated by thoracotomy. But conventional thoracotomy has large trauma, large bleeding volume and long recovery time. Video-assisted thoracoscopic surgery (VATS) has been extensively used for decortication of postpneumonic empyema with good results [3] and several literature reported its utility in tuberculous empyema,especially for Stage III tuberculous empyema [4, 5].

At present, VATS treatment of tuberculous empyema requires 2–3 surgical incisions [6]. Single hole VATS, and the incision is only 2.5 cm,which has rarely been reported in treatment of advanced tuberculous empyema. We report the case of a 23-year-old woman with advanced tuberculous empyema treated by a 2.5 cm single port VATS pleural decortication.

## Case presentation

**Fig. 1:**
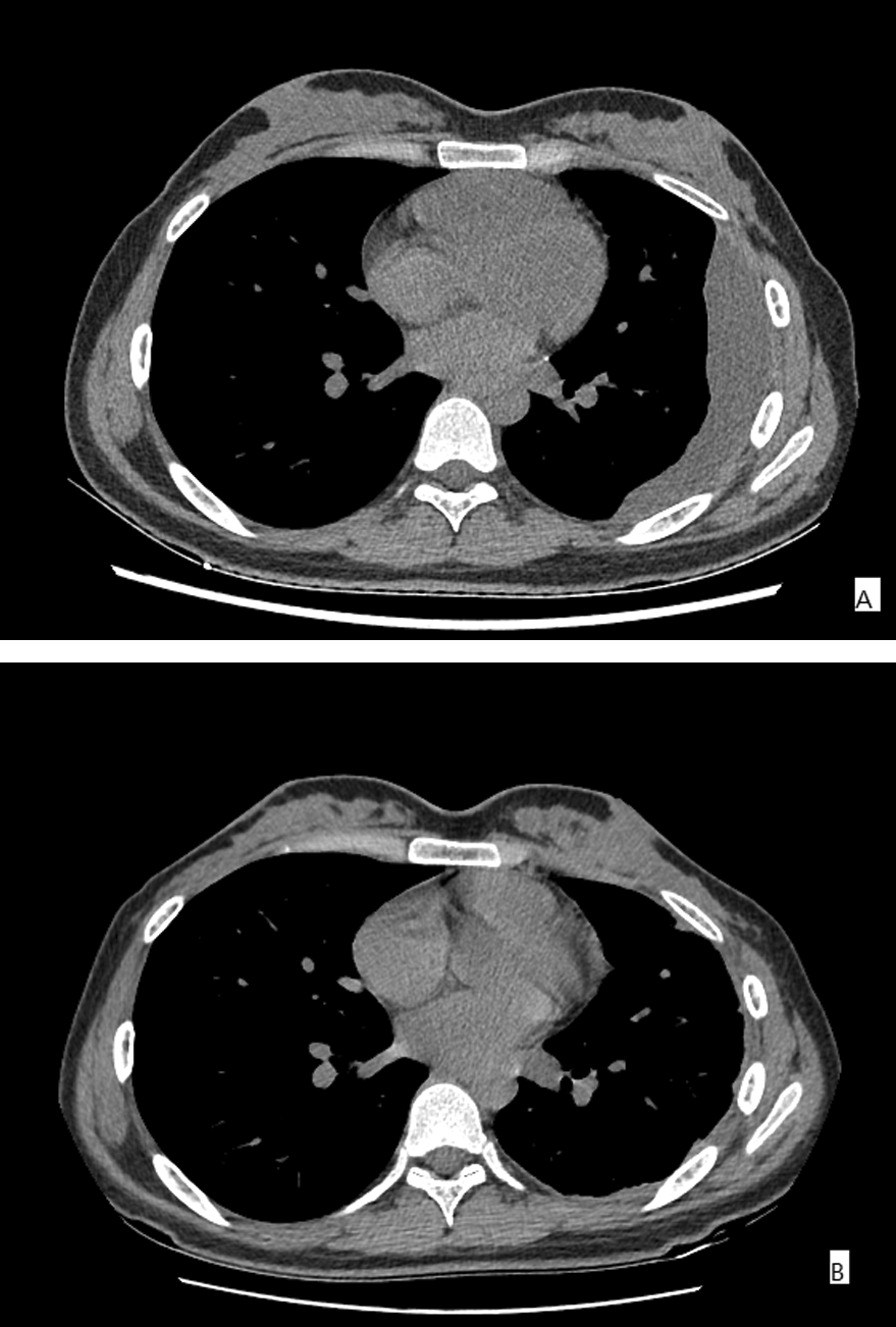
CT scan for the patient with tuberculous empyema. **A** CT for the patient with tuberculous empyema before operation. **B** CT for the patient after surgery for 6 months. *CT* computer tomography

A 23-year-old woman admitted to our hospital with complaints of intermittent chest pain for half a year. Half a year ago, computed tomography scan revealed the left pleural thickening and left pleural effusion, and tuberculous pleural effusion was considered. Thoracic puncture and culture of tuberculosis in pleural effusion suggest tuberculous pleurisy. Standardized anti-tuberculosis treatment with isoniazid, rifampicin, and ethambutol was given in the local community hospital, and then adjusted to isoniazid, rifapentine, ethambutol, levofloxacin after two months. The total course of treatment was 6 months. A reexamination of chest CT revealed that left encapsulated pleural effusion. The maximum diameter of encysted abscess cavity was 10 cm, the thickness of the hypertrophy pleural fiberboard was 2 cm (Fig. 1a). The diagnosis was Stage III tuberculous encapsulated empyema. Considering that the patient was young and unmarried, we decided to perform minimally invasive pleural decortication through a single port. Chest B-ultrasound was performed to draw the border of the abscess cavity on the chest wall before the operation.

**Fig. 2:**
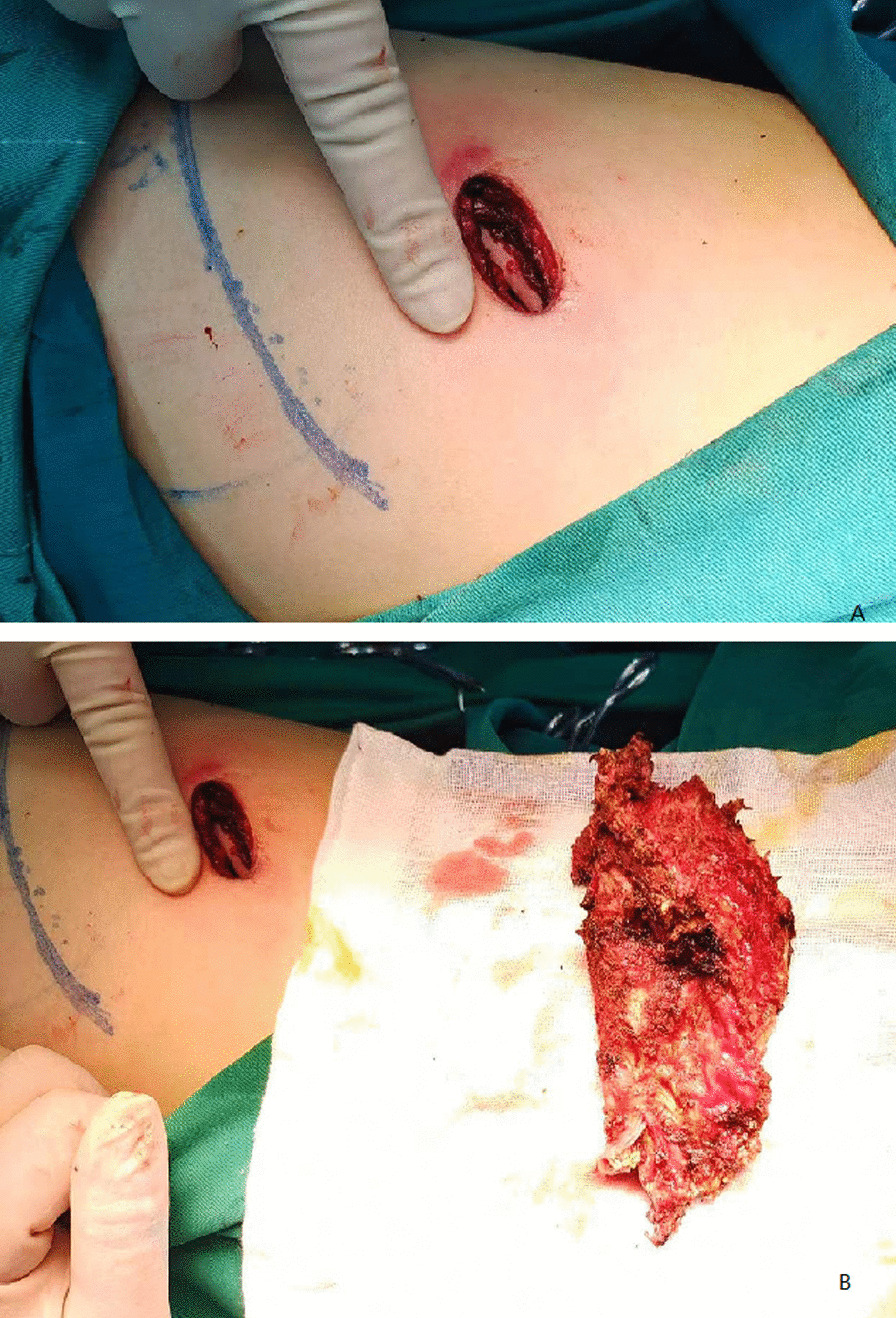
Postoperative surgical would (**A**) and resected abscess cavity (**B**)

A 2.5 cm single port was opened at the eighth intercostal space on the lower margin of the abscess cavity, just anterior to midaxillary line (Fig. 2). After entering the thoracic cavity, the finger was extended into the chest to make appropriate blunt dissection, and a small cavity was expanded. A 10-mm 30° thoracoscope was placed at the posterior side of the incision and other working instruments at the anterior side. First, the visceral peel should be removed from the lung surface using the tip of the suction and electrocautery. Because the operating space is narrow, we bent the coagulation scalpel head to fit the angle of the thoracic cavity. Then, the empyema pouch was suspended on the chest wall, which facilitated the decortication of the empyema pouch. Then the parietal pleura was dissected by electric scalpel close to the inner side of the rib. The margin of the empyema was extrapleural dissection, and the empyema pouch was completely removed. To avoid contamination of the chest cavity, we remove the empyema pouch closed without opening it. before taking out the empyema pouch, the abscess was sucked out by suction to reduce its volume and specimen was collected for various microbiological tests. The chest cavity was repeatedly irrigated with 0.9% warm saline, the leakage position was sutured intermittently. One chest tube was inserted via the port after removal of the instruments. The operation time was 240 min, and blood loss was 100 ml. The patient recovered well and the narcotic analgesics were stopped on postoperative day 1. The anti-TB treatment continued after operation with rifampicin, ethambutol, and isoniazid, along with the continuous use of liver-protective drugs. The patient was followed up without recurrent disease for 6 months (Fig. 1b).

## Discussion

Stage III Tuberculous Empyema is a type of encapsulated empyema which is difficult to deal with. Most of these patients need surgical treatment. Traditionally, thoracotomy had been the preferred surgical approach, which had large surgical trauma and more intraoperative bleeding. Nowadays, with the development of minimally invasive surgery technology, thoracoscopic surgery has also been applied to the treatment of Stage III tuberculous empyema, but most of them need multiple ports [7], and a small number of single port surgery generally need a longer incision of more than 6 cm [5]. Tuberculous empyema often occurs in young patients. In our current case, the unmarried young patient has a high cosmetic needs. So we chose mini single port surgery to treat this patient. In this case, we successfully performed VATS for Stage III Tuberculous Empyema with a mini single port of 2.5 cm in size. During surgery, we apply surgical techniques of empyemectomy.We remove the empyema pouch closed without opening it to decrease contamination of chest cavity and this might be the first report via uniportal VATS.

Before the operation, accurate and comprehensive preoperative examination is very necessary. Ultrasonography is a useful and non-invasive tool for identifying empyema stages, for planning the surgical approach [8]. In our report, we draw the border of the empyema on the chest wall by B-ultrasound before the operation and determine the position of the incision.

There are many difficulties in single port thoracoscopic surgery. When the operation equipments enter the narrow single hole at the same time, there will be mutual influence. The position angle of the lateral chest wall is difficult to reach, so we bent the coagulation scalpel head to adapt to the angle in the thoracic cavity. The upper edge of the lesion is far from the single port, which is difficult to operate. Because of the above reasons, the operation time is long. Some authors removed part of the rib in order to expand the operating space [5]. Although the surgical trauma was increased, the surgical safety was increased, and it was suitable for some patients with heavy adhesions.

Single port VATS pleural decortication has advantages and disadvantages over conventional thoracoscopic or open surgery. First of all, the postoperative pain is significantly reduced due to the small incision and only one intercostal space is involved. Some articles showed that the pain of patients after single-port thoracoscopic surgery was significantly mild [9]. In our case, no additional analgesic drugs were used after operation. Furthermore, most patients with tuberculous encapsulated empyema are young patients, and they also have cosmesis needs for small surgical incision. Our patient, as an unmarried young woman, is satisfied with the results of minimally invasive surgery. Last but not least, Single-port VATS pleural decortication significantly reduces intraoperative blood loss and helps patients recover faster than traditional surgery [10].

The disadvantages of this method originate mainly from utilizing various surgical equipments and the camera through the same small incision, as a coordinated maneuver may be challenging. Secondly, the limitation of exposure can be a disadvantage over open surgery. Thirdly, the distance between the incision and the operation area is far, which makes the operation more difficult. Therefore, there could be some limitations to apply single-port VATS in all patients and in all surgeons. However, on the whole, all these drawbacks can be overcome by increased personal experience of the surgeon.

## Conclusion

In conclusion, single-port VATS could be a useful option among various surgical approaches for Stage III Tuberculous Empyema in well selected patients. On the premise of full examination, we can consider choosing a smaller surgical incision for surgical treatment of Stage III tuberculous empyema. It has less blood loss and less postoperative pain, which is also more in line with the high cosmetic needs of patients.We recommend that single-port VATS with a smaller incision for the Stage III tuberculous empyema should be considered in well selected patients by very experienced surgeons.

## Data Availability

Not applicable.
